# Revealing the genetic complexity of hypothyroidism: integrating complementary association methods

**DOI:** 10.3389/fgene.2024.1409226

**Published:** 2024-06-11

**Authors:** Roei Zucker, Michael Kovalerchik, Amos Stern, Hadasa Kaufman, Michal Linial

**Affiliations:** ^1^ The Rachel and Selim Benin School of Computer Science and Engineering, The Hebrew University of Jerusalem, Jerusalem, Israel; ^2^ Department of Biological Chemistry, Institute of Life Sciences, The Hebrew University of Jerusalem, Jerusalem, Israel

**Keywords:** UK-Biobank, GWAS, Hashimoto’s thyroiditis, open targets, genotyping, PWAS, congenital hypothyroidism, FinnGen

## Abstract

Hypothyroidism is a common endocrine disorder whose prevalence increases with age. The disease manifests itself when the thyroid gland fails to produce sufficient thyroid hormones. The disorder includes cases of congenital hypothyroidism (CH), but most cases exhibit hormonal feedback dysregulation and destruction of the thyroid gland by autoantibodies. In this study, we sought to identify causal genes for hypothyroidism in large populations. The study used the UK-Biobank (UKB) database, reporting on 13,687 cases of European ancestry. We used GWAS compilation from Open Targets (OT) and tuned protocols focusing on genes and coding regions, along with complementary association methods of PWAS (proteome-based) and TWAS (transcriptome-based). Comparing summary statistics from numerous GWAS revealed a limited number of variants associated with thyroid development. The proteome-wide association study method identified 77 statistically significant genes, half of which are located within the Chr6-MHC locus and are enriched with autoimmunity-related genes. While coding GWAS and PWAS highlighted the centrality of immune-related genes, OT and transcriptome-wide association study mostly identified genes involved in thyroid developmental programs. We used independent populations from Finland (FinnGen) and the Taiwan cohort to validate the PWAS results. The higher prevalence in females relative to males is substantiated as the polygenic risk score prediction of hypothyroidism relied mostly from the female group genetics. Comparing results from OT, TWAS, and PWAS revealed the complementary facets of hypothyroidism’s etiology. This study underscores the significance of synthesizing gene-phenotype association methods for this common, intricate disease. We propose that the integration of established association methods enhances interpretability and clinical utility.

## 1 Introduction

Hypothyroidism is a disorder of the endocrine system in which the thyroid gland does not produce enough hormones or when the thyroid hormones act inadequately in target tissues (Almandoz and Gharib, 2012). Hypothyroidism (EFO: 0004705) and its extreme condition, myxedema (EFO: 1001055), are signified by impairment in the function of the thyroid (Chaker et al., 2022). The thyroid gland is crucial to the metabolism of all tissues and the early development of the central nervous system (CNS) (Patel et al., 2011). While over 10% of the world’s population exhibits some level of iodine deficiency that may lead to hypothyroidism, it does not apply to the developed world (Taylor et al., 2018). In the United States, the prevalence of hypothyroidism has been shown to steadily increase over the last two decades, reaching 14.4% (clinical and preclinical) (Wyne et al., 2022). Subclinical hypothyroidism accounts for 4%–8% of the population (Fatourechi, 2009). It is estimated that one in eight people will develop a functional deficiency of the thyroid in their lifetime, with a 3-4-fold higher likelihood of females relative to males (Uzunlulu et al., 2007; Chung, 2014).

Primary hypothyroidism is defined by a failure of the thyroid gland itself. Secondary and tertiary hypothyroidism are caused by dysfunction of the pituitary and hypothalamus glands, respectively (Chaker et al., 2022). The diagnosis of hypothyroidism is determined by free and bound thyroid hormones in the blood, the level of TSH, and the composition of autoantibodies to thyroid markers (Evered et al., 1973). Specifically, autoantibodies against thyroid-specific antigens (e.g., TSHR, TG, and TPO) were found in most patients (Brown, 2013). The majority of these cases can be assigned to hypothyroidism with an autoimmunity component (e.g., Hashimoto’s thyroiditis and autoimmune hypothyroidism, Ord’s thyroiditis) (Eriksson et al., 2012). Importantly, hypothyroidism is linked to a higher incidence of other organ-specific autoimmune diseases (Brown, 2013; Matzaraki et al., 2017). Hyperthyroidism occurs in children in the form of autoimmune thyroiditis (AIT) (Brown, 2013). AIT reflects some unknown defects in immunoregulation, which translate into injury to thyroid tissue, which in turn activates apoptotic cell death and thyroiditis. The genetic basis for AIT is unknown, but it is likely to combine genetics (estimated to account for 70% of the risk for developing AIT) and environmental factors that interact with predisposed genetics (Brent, 2010). An interest in thyroid function in adults and especially in the elderly relies on the increasing links between thyroid status and cognitive function, cardiovascular diseases, healthy aging, and longevity (Aggarwal and Razvi, 2013). It is imperative to identify people at higher risk and tune clinical treatment to avoid negative impacts on quality of life (Hegedüs et al., 2022). Moreover, the understanding of the environmental factors that contribute to disease development is limited, and risk factors may include hormones (e.g., estrogen), stress, smoking, and dietary iodine consumption.

Hyperthyroidism may also be congenital, where the incidence rate is one in every 2000–4,000 live births. Congenital hypothyroidism (CH) is a developmental abnormality affecting the hypothalamic-pituitary-thyroid (HPT) axis (Persani et al., 2018). Primary CH, which is associated with a missing or underdeveloped thyroid (dysgenesis), is the most common neonatal disease and accounts for most CH. Most cases of CH occur sporadically and are frequently associated with an increase in neonatal malformations, which can result in further complications (Léger et al., 2014). Unfortunately, the genetics of thyroid dysgenesis are resolved in only 5% of cases (Wassner, 2020). A systematic CH screen in Japanese (Narumi et al., 2010) and Czech (Al Taji et al., 2007) individuals confirmed the challenge of identifying causal mutations. While the most pathogenic variants of the TSH receptor (TSHR) are nonsyndromic, mutated Gsα (GNAS1) and PDE8B, which are components of TSHR signaling, are linked with syndromic disease (Persani et al., 2010). Candidate genes that potentially disrupt thyroid gland formation have been linked to other rare monogenic diseases [reviewed in (Stoupa et al., 2021)]. Mutations in numerous thyroid transcription factors (TITF-1, TITF-2, PAX-8, FOXE1, GLIS3) are mostly syndromic (Kostopoulou et al., 2021). Additionally, mutated genes that act in the biosynthesis and cell biology of thyroid hormones (Panicker, 2011) may cause dysfunction of thyroid hormone synthesis and secretion (dyshormonogenesis). Among these genes are thyroid peroxidase (TPO), thyroglobulin (TG), sodium iodide symporter (NIS), pendrin (PDS), thyroid oxidase 2 (THOX2), and iodotyrosine deiodinase (IYD). The iodothyronine transporter (MCT8), which is expressed in the thyroid gland membrane, was also shown to drive hypothyroidism, which is coupled to neurological deficits (Park and Chatterjee, 2005). Dyshormonogenetic cases are often recessively inherited (Makretskaya et al., 2018). Interestingly, the occurrence of mutated CH causal genes differs substantially across populations (Sun et al., 2018).

In this study, we analyzed the genetic signatures among people diagnosed with ICD-10 E03 (“other hypothyroidism”), with most patients (98%) being diagnosed with E03.9 (hypothyroidism, unspecified) that includes patients with primary, secondary, and tertiary hypothyroidism in the UKB. We asked whether the genetic effects of hypothyroidism and myxedema are associated with sex. To this end, we applied several association methods, most notably the proteome-wide association study (PWAS) method, which detects gene-phenotype associations through the effect of variants on protein function (Brandes et al., 2020). By comparing results from the PWAS (Brandes et al., 2020), TWAS (Luningham et al., 2020), classical GWAS, and coding GWAS, we shed light on the complex etiologies of hypothyroidism with or without an immunological basis. We conclude that the integration of established association methods and partitioning the population by sex can improve interpretability and clinical utility.

## 2 Materials and methods

### 2.1 UKB processing

The UK Biobank (UKB) is a population-based database with detailed medical, genotyping, and lifestyle information covering ∼500 k people aged 40–69 across the UK who were recruited from 2006 to 2010. The analyses herein were based on the 2019 UKB release. We restricted the analysis to European origin (codes 1, 1001, 1002, and 1003, respectively; and ethnic background, data field 21000). We applied the classification according to genetic ancestry (Genetic ethnic group, data field 22006). We further removed genetic relatives by randomly keeping only one representative of each kinship group.

Hypothyroidism is indexed by ICD-10 code E03. The analysis includes individuals who have any diagnosis within the main or secondary codes (UKB data fields 41,202 and 41,204, respectively) and the summary diagnosis code 41270. The latter covers the distinct diagnosis codes a participant has recorded across all their hospital inpatient records, in either the primary or secondary position. These fields cover ICD-10 from E03.0 to E03.9 (total 29,478 participants), with 98.5% of them marked as “unspecified hypothyroidism”, “other specified hypothyroidism” (E03.8, 0.7%), “hypothyroidism due to medicaments and other exogenous substances” (0.3%, E03.2), and CH (0.3%, E03.0-E03.1). We included “other hypothyroidism” and excluded “iodine-deficiency-related hypothyroidism” (E00-E02) and postprocedural hypothyroidism (E89.0). This set of E03 with genotyping data includes 2,557 males and 11,094 females.

### 2.2 All GWAS and coding GWAS analyses

We processed data from the UKB as described above to perform all GWAS and coding GWAS. UKB released genotyped data for all participants. In genotyping data, there are ∼820 k preselected genetic variations (UKB Axiome Array). Based on the UKB imputation protocol, the number of variants was expanded to 97, 013, 422. For the imputed variants, we calculated the probabilistic expectations for the alternative alleles (Brandes et al., 2020). We applied a standard PLINK protocol to filter candidate variants for the analysis. For the gene length (g-GWAS), we considered variants with a MAF threshold of 0.001, a Hardy-Weinberg equilibrium (HWE), exact test *p*-value of 1e-6, and genotyping coverage with a 90% call rate (using the Geno option of 0.1). Altogether, we analyzed 10,258,628 variants. We also included as covariates sex, year of birth, and the first six principal components (PCs) to account for population structure. We considered variants within genes (exons and introns) as indicators of gene association. However, we have not discussed variants located at other functional regions beyond the RefSeq transcript of coding genes. For E03 GWAS, we had 12,435 cases and 257,948 controls.

The coding GWAS analysis includes the human proteome according to UniProt-SwissProt (labeled “reviewed”). Due to the unambiguous mapping of RefSeq gene names, we cover 18,053 protein-coding genes of the ∼20 k proteins that are listed for the human proteome (see Supplementary Material S1). For all GWAS and coding GWAS included 172 covariates that include sex (binary), year of birth (numeric), 40 principal components (PCs) that capture ancestry stratification (numeric), the UKB genotyping batch (one-hot-encoding, 105), and the UKB assessment centers associated with each sample (binary, 25).

### 2.3 GWAS summary statistics

We used the Open Targets Genetics (OTG) platform to select current knowledge and GWAS results on hypothyroidism (Carvalho-Silva et al., 2019). The OTG (release date: 6/2023) unifies multiple sources of evidence for an inclusive list of 2007 genes, each ranked by an OT global score (range 0–1.0). Among these genes, 702 genes are supported by genetic association (GA) scores based on large-scale independent GWAS summary statistics (Carvalho-Silva et al., 2019) (see Supplementary Material S1). Other datasets from the OTG platform include “permanent congenital hypothyroidism” with 53 associated genes, 35 of which have a GA score >0.5. This phenotype is a merger of Orphanet: 442 (23 genes) and EFO 0016408. Most of the associated genes were derived from ClinVar and Orphanet (Sharo et al., 2023).

### 2.4 PWAS functional effect score per gene of the human proteome

The PWAS methodology assumes that causal variants in coding regions affect phenotypes by altering the biochemical functions of the encoded protein of a gene. In summary, the functional impact rating at the molecular level (FIRM) from the pretrained machine-learning (ML) model is then used to estimate the extent of the damage caused to each protein in the entire proteome (Brandes et al., 2019). FIRM performance was reported and validated for the pathological variants in ClinVar, reaching an AUC of 90% and accuracy of 82.7% (Brandes et al., 2019). The predicted effect score of a variant is a number between 0 (complete loss of function, LoF) and 1 (no functional effect, synonymous variant). PWAS explicitly treated in-frame indels (Brandes et al., 2020). We seek a calibrated score for the overall protein damage at an individual level. Thus, per-variant damage predictions are aggregated at the gene level according to recessive, dominant and hybrid gene heritability modes. On average, there are 35.4 nonsense and missense mutations per gene that are considered for the gene-based effect score. PWAS results are based on the same set of variants as used for the coding GWAS, i.e., 639,323 variants located within 18,053 protein-coding genes and 172 covariates.

### 2.5 Transcriptomics association studies (TWAS) analysis

We used the webTWAS database (Cao et al., 2022), which integrates publicly available GWAS summary data with transcriptomics association (TWAS) models. We used TWAS for hypothyroidism/myxoedema based on 22,141 cases and 452,264 controls of European origin (Roslin Institute, Study AT034). We utilized the UTMOST model with cross-tissue expression imputation (Hu et al., 2019). UTMOST uses a multi-tasking learning method to impute gene expression in 44 human tissues simultaneously. Combination of multiple single-tissue association scores into a joint-tissue test is expected to improve the quantify of gene-disease association. Notably, TWAS provides a rich collection of models for disease associations with genomic loci (Gusev et al., 2016). Often, a single locus is associated with many genes (e.g., >10 genes/locus), therefore, TWAS may suffer from an expansion in gene candidates. We have not explored the coherence between the different TWAS models.

### 2.6 Validation scheme

An independent cohort of FinnGen was used for the validation of genes identified as statistically significant by the PWAS method (ICD10: E03). The analysis is based on a recent version of FinnGen with ∼350,000 individuals with no overlap with UKB participants (Kurki et al., 2023). For additional details on FinnGen validation, see Supplementary Material S1. We further extended the validation according to GWAS analysis from Taiwan with about 2,700 cases and ∼32,000 controls (Traglia et al., 2017). Interestingly, as many of the associated variants were associated with non-coding RNA, antisense and pseudogenes, we filtered the data to associated variants within coding genes (824 variants).

### 2.7 Statistical tests

#### 2.7.1 Effect size statistics

To determine the effect size of a gene on hypothyroidism, we applied a measure of Cohen’s d values. Cohen’s d, also known as standardized mean difference, measures the difference between two means divided by a standard deviation (SD) for the data. In this study, Cohen’s d is the (normalized) difference in mean gene effect scores between cases and controls (calculated independently for both dominant and recessive effect scores). For GWAS, the variant association and effect size were calculated by PLINK 2.0 default logistic regression, which produces the z score to specify the effect size and its directionality. Note that in GWAS, a positive z score indicates a positive correlation between hypothyroidism and the number of alternative alleles, thereby indicating a risk variant. In PWAS, positive values indicate a positive correlation with the gene effect scores, whose higher values mean less functional damage. Thus, negative values are indicative of protective variants in GWAS *versus* risk genes in PWAS.

#### 2.7.2 PRS calculation

We applied a procedure for assessing the possibility that the difference in the UKB cohort sizes (2,557 males and 11,094 females) is due to sex-specific effects. To this end, we calculated PRS by the PRSice-2 protocol (Choi and O'Reilly, 2019). Predictive PRS models for coding GWAS, and all GWAS were based on a standard partition of 80:20 for the training and test sets. For all GWAS, we used 10.2 M common variants (MAF >1e-03, a *p*-value for HWE test larger than >1e−06 and 90% call rate using geno option). In addition, we applied covariates of sex, age, UKB assessment centers, and genotype measurement batch. We performed predictive PRS for hypothyroidism E03 by the liability scale *R* ^2^ and the AUC-ROC (i.e., the area under the receiver operating characteristic curve) (Choi and O'Reilly, 2019) for both sexes, male and female groups. While the *R* ^2^ assesses the amount of explained variation in the regression models, the AUC-ROC evaluates the ability of the set of used variants to discriminate between the classes (E03 vs. controls).

### 2.8 Bioinformatics tools

For gene connectivity and protein‒protein interaction (PPI) maps, we applied STRING at a high PPI connectivity score (Szklarczyk et al., 2021). For functional enrichment of GO annotation and KEGG pathways, we applied the Gene2Func function of FUMA-GWAS using default parameters and a set of genes as input (Watanabe et al., 2017). All values are reported by their adjusted *p*-values, using the human proteome as background.

### 2.9 Resource and availability

FIRM model and prediction of variant-centric effect score (https://github.com/nadavbra/firm). The PWAS is available in https://github.com/nadavbra/pwas. Exclusion and inclusion rules per outcomes and phenotypes from FinnGen are found in https://risteys.finngen.fi/endpoints/. For summary statistics GWAS comparison we utilize the compilation from Open Targets Genetics (OTG) with https://genetics.opentargets.org/study-comparison/NEALE2_20002_1226 as an anchor. All supporting data is provided in Supplementary Material S1, Supplementary Material S2: Supplementary Tables S1–S10, Supplementary Material S3: Supplementary Figures S1–S3.

## 3 Results

### 3.1 Comparative GWAS results for hypothyroidism

**FIGURE 1:**
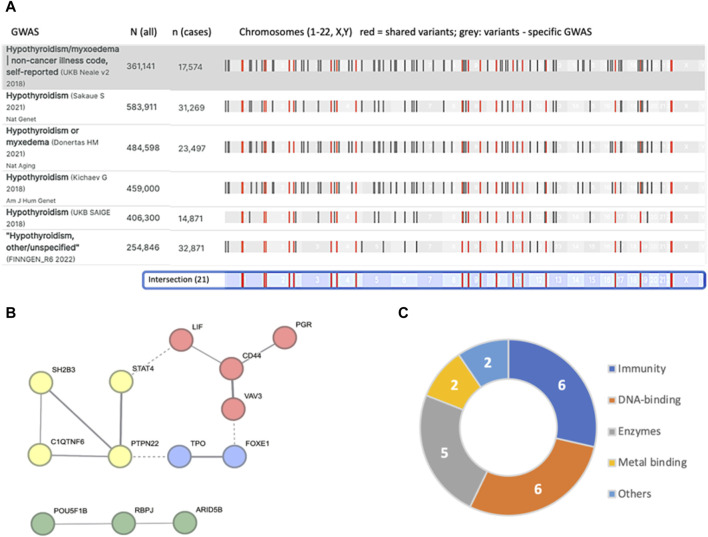
Summary of independent loci identified from major GWAS results as compiled in the OTG portal. **(A)** The number of participants in each study and the number of hypothyroidism cases are indicated by N (all) and n (cases). There are 21 variants that are shared by all six studies (colored red). The chromosomal position is shown (bottom, light blue). **(B)** STRING analysis of the 21 mapped associated genes resulted in a network of 13 genes (interaction score >0.4). The nodes are colored by PPI clusters. Evidence of connectivity between the clusters is indicated by dashed lines. **(C)** Connectivity of the 21 associated genes (Table 1) and their major functional annotations.

Large-scale GWAS that was performed on several cohorts for hypothyroidism (see Methods) is compiled in the Open Targets Genetics (OTG) platform. A comparative study compiling six of the largest studies is shown in Figure 1. The comparison is performed with a GWAS of “Hypothyroidism/Myxoedema (noncancer, self-reported)” from Neale v2, 2018 that covers non-Finnish Europeans with ∼17.5 k cases and ∼345 k controls from UKB. This study reports on 115 significant (*p*-value <5e-8) variants. Each of the leading variants is reported along with its most likely associated genes.

We identified 21 intersecting lists for all 6 GWAS (Supplementary Material S3: Supplementary Table S2). Note that the individual GWAS may include overlapping participants. While accurate mapping of variants to genes is inconclusive, we observed significant functional connectivity among these overlapping associated genes (STRING PPI enrichment *p*-value of 7.4e-07; Figure 1B). For example, the TPO and FOXE1 genes (Figure 1B, blue cluster) are involved in thyroid hormone production and secretion. Specifically, TPO is a key enzyme in thyroid peroxidase that acts in the iodination of tyrosine residues in thyroglobulin and thyroid hormones, while FOXE1 is implicated in thyroid gland morphogenesis (Table 1). The clusters in Figure 1B list genes active in the regulation of T-cell receptors (yellow), thyroid hormone production (blue), transcriptional regulation (red), and chromatin modifiers (green). We observed that the genes associated with hypothyroidism partition genes by their cellular properties in relation to immunity, DNA-binding proteins, and numerous enzymes (Figure 1C).

**TABLE 1:** The intersection variants (total 21) from six large-scale hypothyroidism GWAS results.

#	SNP rsID	Band	Variant	Closest gene	AF^a^	# LD genes^b^
1	rs78765971	1p13	1_107819547_GAC_G	VAV3	0.09	3
2	rs484959	1p13	1_109823461_T_C	GSTM3	0.50	23
3	rs2476601	1p13	1_113834946_A_G	PTPN22	0.89	15
4	rs11675342	2p25	2_1403856_C_T	TPO	0.44	3
5	rs1534430	2p24	2_12504610_C_T	TRIB2	0.41	1
6	rs2111485	2q24	2_162254026_A_G	FAP	0.62	6
7	rs7582694	2q32	2_191105394_C_G	STAT4	0.78	7
8	rs76897057	3q28	3_188407079_TA_T	LPP	0.48	1
9	rs34046593	4p15	4_26109971_G_A	RBPJ	0.31	6
10	rs546532456	4q31	4_148724495_C_CTT	PGR	0.19	1
11	rs2445610	8q24	8_127184843_A_G	POU5F1B	0.35	2
12	rs2123340	9p21	9_21589042_G_A	IFNE	0.65	19
13	rs7850258	9q22	9_97786731_A_G	FOXE1	0.66	14
14	rs71508903	10q21	10_62020112_C_T	ARID5B	0.18	4
15	rs736374	11p13	11_35245397_G_A	CD44	0.37	7
16	rs4409785	11q21	11_95578258_T_C	SESN3	0.17	5
17	rs3184504	12q24	12_111446804_T_C	SH2B3	0.52	17
18	rs61759532	17p13	17_7337072_C_T	ACAP1	0.23	59
19	rs10424978	19p13	19_4837545_C_A	TICAM1	0.59	21
20	rs145429422	22q12	22_30125266_CCAG_C	LIF	0.48	23
21	rs229540	22q12	22_37195250_T_G	C1QTNF6	0.42	25

^a^ AF, Allele frequency for non-Finnish European population.

^b^ # LD genes, the number of associated mapped genes resulting from the variant to gene (V2G) OTG, protocol.

Table 1 summarizes the variants (based on the overlap of six large-scale GWAS, Figure 1) along with the most likely associated genes. Most variants are common, with allele frequencies (AFs) ranging from 0.17 to 0.89. Note that for many of the variants, linkage disequilibrium (LD) identifies a large number of genes within the same haplotype block. In these cases, no conclusive assignment to a particular gene is possible without fine mapping. In fact, only 3 of the 21 lead variants are associated with a definitive gene (Table 1).

The listed shared variants are quite stable and remain valid in view of additional large-scale GWAS. For example, addition of GWAS for autoimmune thyroid disease with 755 k participants from Iceland (93 associated variants) (Saevarsdottir et al., 2020) had only a minor influence on the overall number of intersected variants (19 of 21 listed variants shared by all 7 studies). Under the assumption of accurate mapping of variants to genes (Table 1), the results expose the genetic signal of CH. Specifically, TPO was reported as causal for thyroid dyshormonogenesis 2 (OMIM 274500). In the Chinese population (Wang et al., 2017), abnormal expression of FOXE1 was linked to CH-based thyroid dysgenesis (OMIM 218700). Similarly, polymorphisms in the listed genes VAV3, SH2B3, FOXE1 and PTPN22 were identified in the 23andMe database to be associated not only with hypothyroidism but also with other autoimmune diseases (Eriksson et al., 2012). We conclude that the shared GWAS results identified pleiotropic effects of genes involved in autoimmunity and gene developmental alterations that underlie CH.

### 3.2 GWAS enriched with genes within the MHC extended locus

**FIGURE 2:**
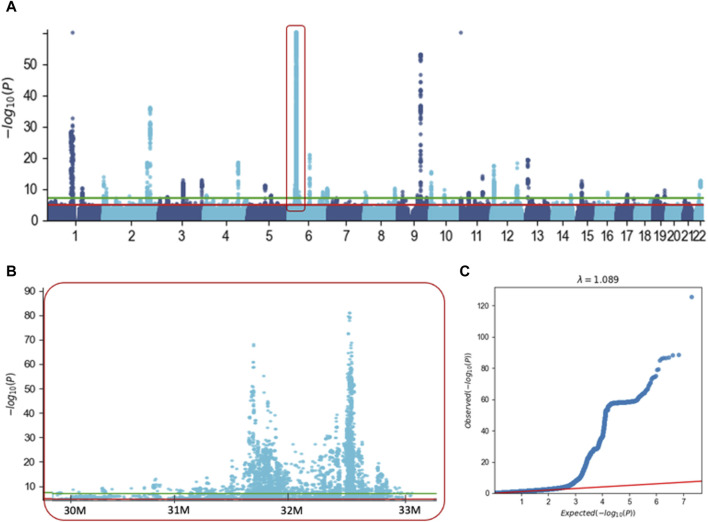
GWAS results for hypothyroidism (ICD-10, E03) with ∼10.2 M variants. **(A)** Manhattan plot covering Chr. 1 to Chr. 22. For visualization clarity we capped the *p*-value at <1e-60. Red frame indicates the MHC extended locus on Chr6. **(B)** Zoom in of the Manhattan plot covering part of the extended region of MHC from Chr 6. The significant threshold of 5e-05 and 5e-08 are marked by the red and green horizontal lines, respectively. **(C)** Quantile-quantile (Q–Q) plot based on the results of GWAS using 10.2 M variants. The red line shows that there is no signal in the data, the inflation factor l = 1.089.

We performed GWAS on UKB for ICD-10 E03 and considered 10.2 M variants across the entire genome (Figure 2). Results from GWAS were partitioned into variants that are positioned within the position of coding genes (refer to g-GWAS) and the rest of the variants that are intergenic. Among the 21,127 associated variants with a *p*-value <5e-05, 81% are intergenic. The rest of the analysis was restricted to the 10,583 associated variants that met the accepted threshold of significance (*p*-value <5e-08, Figure 2A, green horizontal line).

These variants are partitioned into intergenic regions (87%) and variants are located within genes (1,409 variants, assigned to 134 genes, Supplementary Material S2: Supplementary Table S3). Importantly, the intergenic variants were clustered at 32 loci (each <1M, Figure 2A), with an exceptionally significant number of variants in chromosome 6. Actually, 86% of all variants were in the extended region of MHC spanning ∼6 M in chromosome 6 (Chr6: 27.5 M–33.5 M, Figure 2B). A similar trend was also applied to variants located within gene length (g-GWAS), where the majority (58%) of the associated variants are located within the extended MHC region (Chr6: 27.5 M–33.5 M). Figure 2C shows the QQ plot for the expected and observed statistical values associated with all 10,583 associated variants (*p*-value <5e-08). The significant deviation from the expected line supports the view that there is a strong genetic basis for hypothyroidism. We conclude that most GWAS-associated variants are located in the gene-dense immunological region within the MHC locus.

### 3.3 Coding GWAS highlights the abundance of genes in the MHC extended locus

**FIGURE 3:**
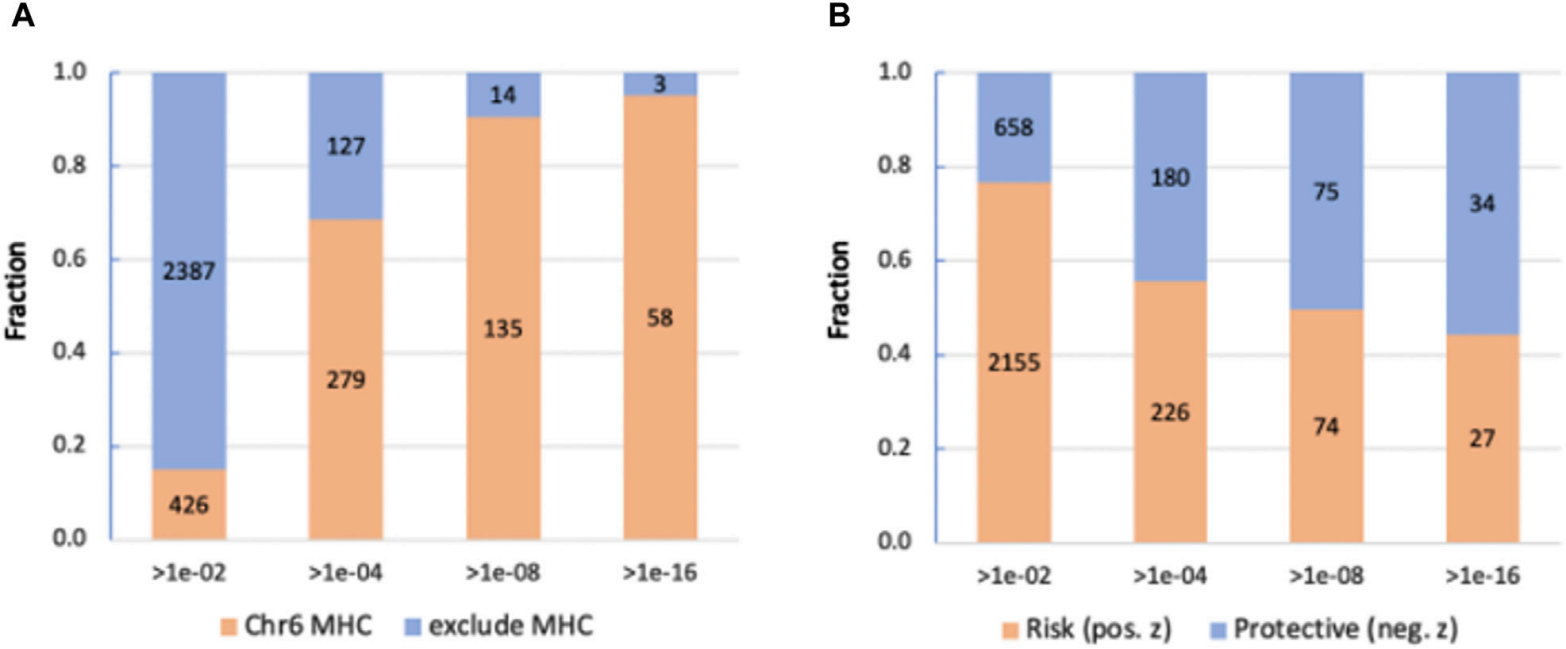
Partition of the significant coding GWAS variants at different thresholds. **(A)** Position of the variants in the Chr6 MHC locus and in other locations. We consider the MHC locus to span 6 M based in the MHC region of Chr6. **(B)** Partition according to the trend of variants that are protective or increase the risk for hypothyroidism.

GWAS results (Figure 2) supported the importance of a gene view for functional interpretation and to overcame the difficulty of variant-to-gene mapping. We have performed GWAS on the coding region using ∼640 k coding variants. Figure 3 shows the results of the analysis for ∼18 k coding genes (Supplementary Material S2: Supplementary Table S4). We report 2813 variants with a relaxed *p*-value of <1e-02 and 406, 149 and 61 variants by setting the significant thresholds for *p*-values of 1e-04, 1e-08 and 1e-16, respectively. Importantly, the fraction of variants associated with the extended region of MHC in chromosome 6 (Chr6: 27.5 M–33.5 M) increased as the *p*-value was more significant, reaching 95% of the significant variants at *p*-value <1e-16 (Figure 3A). The 61 most significant variants are associated with 19 unique genes from the MHC locus and only three genes from other chromosomal locations (Supplementary Material S2: Supplementary Table S4). As the statistical thresholds became more significant (from *p*-value <1e-02 to <1e-16), a shift towards a larger fraction of variants that decrease the risk for hypothyroidism was recorded (Figure 3B).

We conclude that the coding gene view is driven by the signature within the gene-dense immunological region of the MHC locus with the effects of genes on hypothyroidism is bidirectional with equal importance for reducing or elevating the risk.

### 3.4 Gene-based analysis using PWAS

**FIGURE 4:**
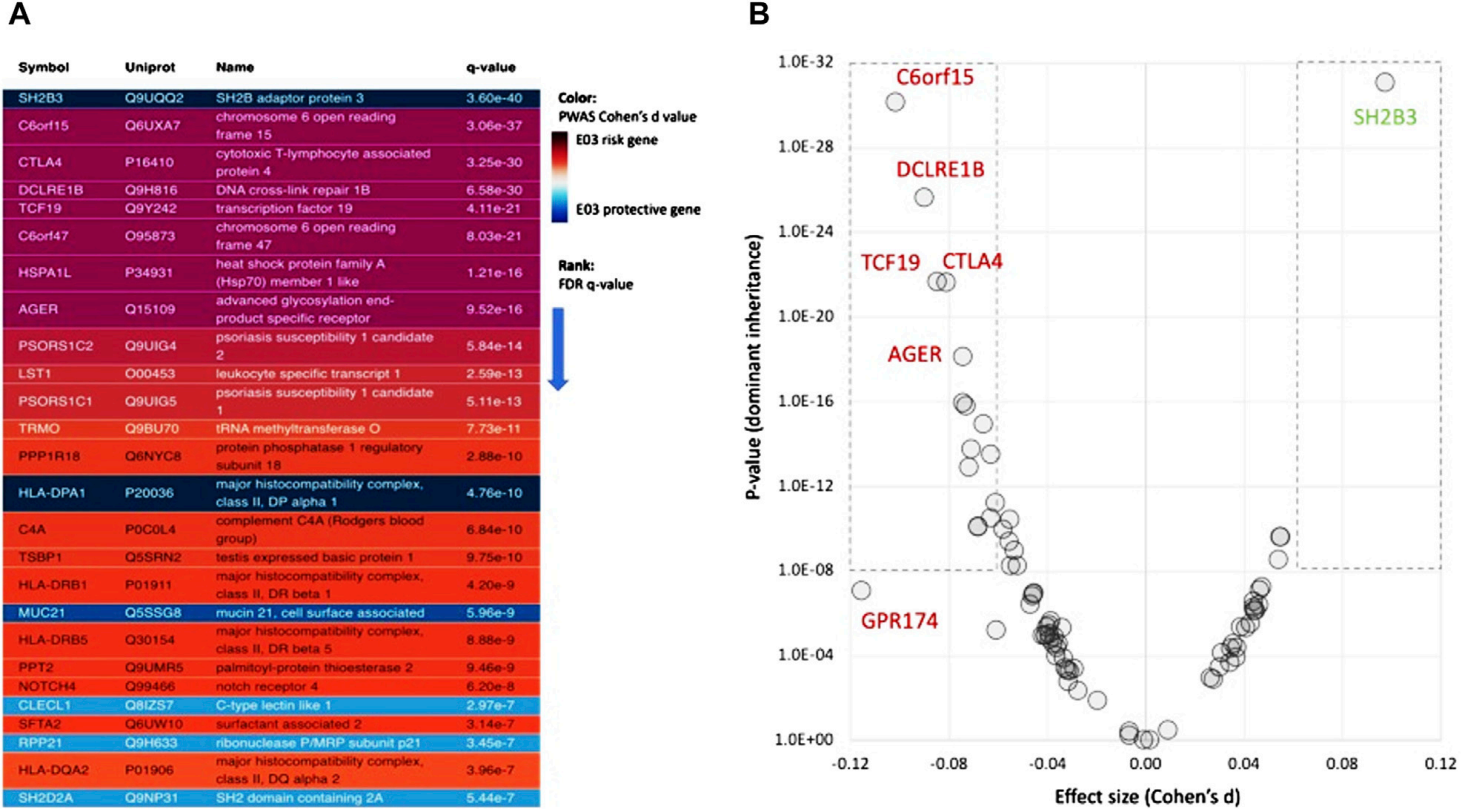
Associated genes from PWAS results. **(A)** Statistically significant genes from PWAS for ICD-10 E03 with q-value <1e-07 (total 26 genes). Genes with an increased and decreased risk are colored purple/red and blue, respectively. **(B)** Effect size (Cohen’s d) for PWAS results for the dominant model. The genes within the dashed frames are associated with Cohen’s d >|0.06|. Positive (green font) and negative (red font) Cohen’s d values are associated with reduced and increased risk, respectively. Supplementary Material S2: Supplementary Table S5 lists all genes and their statistics.

The majority of GWAS results are intergenic (Supplementary Material S2: Supplementary Table S3), and the identified variants within the coding regions (c-GWAS protocol) are independent of each other. To overcome this limitation, we applied PWAS as a gene-based method. PWAS exclusively focuses on alterations in the coding gene and assesses the impact of damaging variants on the protein biochemical function (Brandes et al., 2020). Based on the UKB cohort for ICD-10 E03, we identified 77 statistically significant PWAS genes (FDR-q-value <0.05). We analyzed significant genes based on their risk directionality (Figure 4). Among the top-range genes (FDR q-value, <1e-07; 26 genes, Figure 4A), genes with increased risk for hypothyroidism (colored red) dominate.

As expected from other complex diseases, most genes have a rather limited effect size (calculated by Cohen’s d values). There are six genes that have Cohen’s d values >|0.06| and *p*-values <1.0e-16 (Figure 4B). Among these genes, five genes are associated with elevated risk, and SH2B3 is a strong protective gene. A large effect size is associated with GPR174, G protein-coupled receptor 174, a ChrX gene that plays a role in autoimmunity pathogenesis (Napier et al., 2015). PWAS also model genes according to their inheritance modes. While for 53%, compelling evidence suggests dominant inheritance, 12% of the genes show clear recessive inheritance (Supplementary Material S3: Supplementary Figure S1).

### 3.5 Overlapping genes by complementary genetic association methods

**FIGURE 5:**
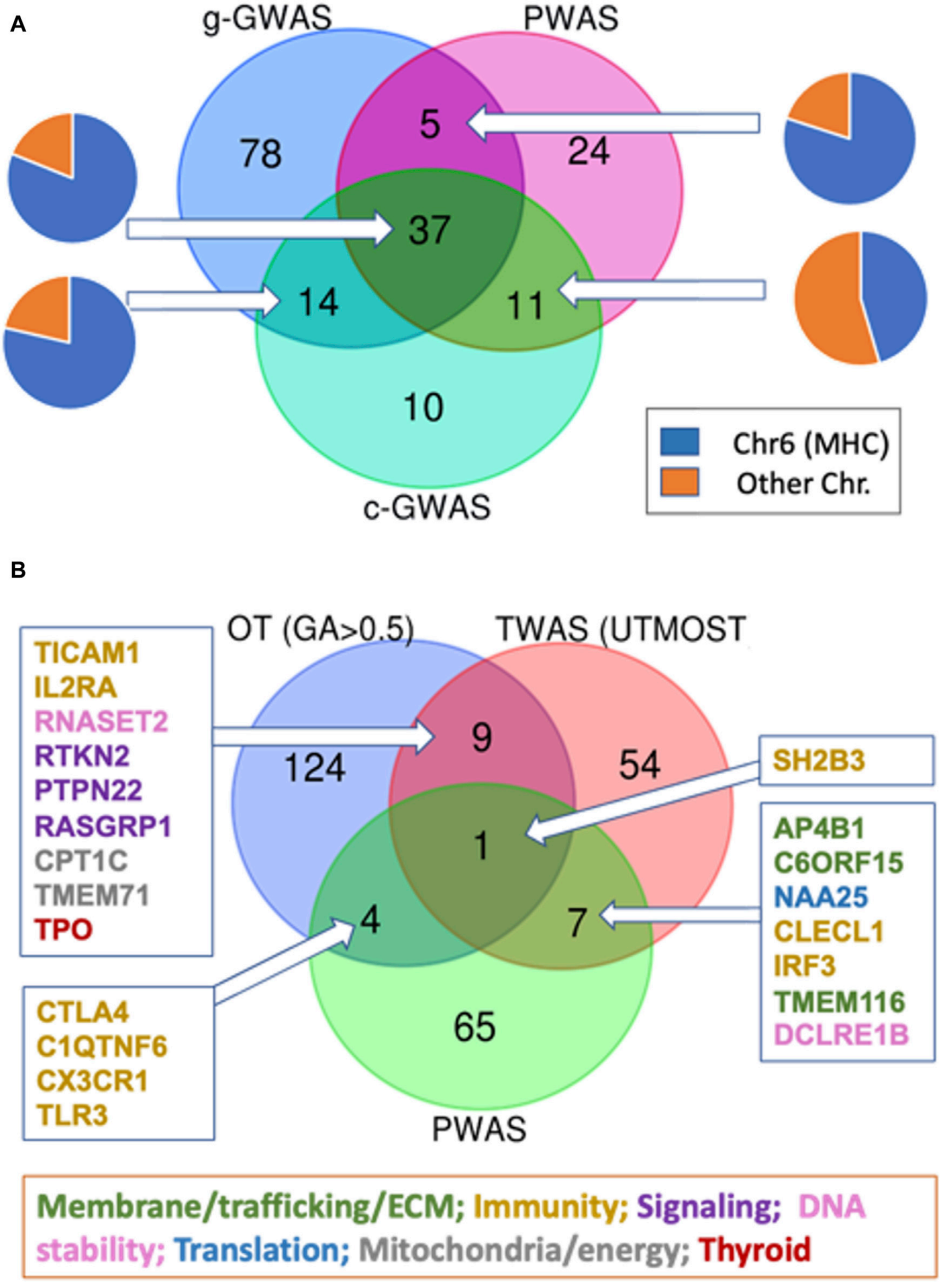
Venn diagram for the overlapping genes according to multiple association studies protocols. **(A)** Venn diagram of gene-length GWAS (g-GWAS, 134 genes), coding GWAS (c-GWAS, 72 genes with *p*-value <5E-07) and PWAS. Each of the overlap section is shown by label the gene as part of the MHC locus (blue) or others (orange). **(B)** Venn diagram of PWAS (77 genes), GWAS (OT, by genetic association score >0.5 (138 genes), and transcription-based association study (TWAS, see Methods) for hypothyroidism/myxedema by UTMOST model (71 genes; Supplementary Material S2: Supplementary Table S7). The subsets of overlapping genes are color-coded according to their main functional annotations.

PWAS method explicitly considers (using FIRM, see Methods) the degree of damage to the protein biochemical function caused by the observed variants per each individual. The scores per each gene are then assessed for significance in a case-control setting for hypothyroidism. PWAS and coding GWAS utilizing the identical variants set in a case-control setting. It is often the case that no single variant is significant in GWAS (e.g., negligible effect size), but following gene aggregation by PWAS the relevance of a gene as a disease-candidate can be confirm (Brandes et al., 2020). We compared the list of significant genes from PWAS and two versions of GWAS (gene-length and coding GWAS) (Figure 5A). Most PWAS genes (69%) overlap with either gene length GWAS or coding GWAS (g-GWAS and c-GWAS, respectively). Inspecting the nature of the overlapping sets between the three tested association methods confirmed the dominant fraction of genes from the MHC locus (Figure 5A, pie charts).

As the vast majority of associated variants occur in noncoding regions (Edwards et al., 2013), we tested the genetic signal that resulted from the transcriptome-wide association study (TWAS) (Luningham et al., 2020). Figure 5B emphasizes the overlap of association studies for hypothyroidism that relied on UKB entries of European origin: the PWAS (77 genes), the external GWAS subset from Open Targets (OT) filtered by genetic association (GA) score >0.5 (138 genes, Supplementary Material S2: Supplementary Table S6), and the TWAS significant expression-trait associations of hypothyroidism (71 genes, Supplementary Material S2: Supplementary Table S7). We show that the gene overlap between PWAS and OT or between TWAS and OT is limited (Figure 5B). Only a few of the overlapping genes between the OT list and TWAS compilation are enriched with cellular immunity genes. Surprisingly, the UTMOST model from TWAS reports that only 4% of the associated genes belong to the MHC locus (3 genes; Supplementary Material S2: Supplementary Table S7). Instead, the overlapping genes belong to diverse aspect of cellular biology including transcription, signaling, trafficking, translation, DNA stability and more (Figure 5B).

Only SH2B3 (SH2B Adaptor Protein 3) was shared by all three orthogonal association studies. Notably, SH2B3 is involved in a range of signaling activities by cytokine receptors and was implicated as a pleiotropic gene. In addition to SH2B3, only four additional overlapping genes between the PWAS and OT lists were identified (Figure 5B). These genes play a role in innate immunity, but none are located within the MHC locus. For example, CTLA4 (cytotoxic T-lymphocyte associated protein 4; Chr 2q33.2) with a missense mutation in rs231775 was also implicated in the autoimmune alopecia areata disease. The C1QTNF6 gene is known to carry two coding mutations, rs229527 (22:37,185,445:C,A) and rs229526 (22:37,185,382:G,C), that are associated with hypothyroidism-related phenotypes. This gene was identified within a locus that is associated with a large number of thyroid-related pathologies (Supplementary Material S3: Supplementary Figure S3). The list of genes from the Venn diagrams is compiled in Supplementary Material S2: Supplementary Table S8).

### 3.6 Validated hypothyroidism PWAS significant genes

To further validate the findings from gene-based PWAS method, we sought an independent population that could validate the gene discovery. To this end, we investigated the Finnish Biobank (FinnGen). Recall that there is no overlap between UKB and FinnGen participants (see Methods). Table 2 lists nine genes (DCLRE1B, CTLA4, TLR3, HLA-DPB1, TRMO, PCSK7, SH2B3, THOC5, and C1QTN) that were validated from the FinnGen data and shared with the PWAS discovery. Notably, PWAS only refers to coding variants, while like any standard GWAS, FinnGen identifies mostly noncoding variants (Table 2).

**TABLE 2:** Validation of the PWAS gene by FinnGen Fz7 for Hypothyroidism phenotypes.

Symbol	FG^a^ (a,b,c)	*p*-value	rsID	Chr (M)	Gene effect	AID^b^	Pub^c^	More evidence	Risk^d^
C1QTNF6	a,b,c	8.7E-19	rs229541	22:37	Intergenic	+	Frederiksen et al. (2013)	Fine-map 23&me	I
CTLA4	a,b,c	1.1E-44	rs3087243	2:203	missense	+	Braun et al. (1998)	Fine-map 23&me	I
DCLRE1B	a	2.0E-10	rs12127377	1:113	intron	+	Ban et al. (2010)	Japan	D
HLA-DPB1	a,b,c	4.8E-31	rs9277535	6:32	downstream	+	Huang and Jap (2015)	Taiwan	D
PCSK7	a,b,c	8.5E-11	rs76169968	11:117	intron	-	Donertas et al. (2021)		D
SH2B3	a,b,c	3.3E-55	rs7310615	12:111	intron	+	Auburger et al. (2014)	23andMe	D
THOC5	a	2.5E-06	rs8140060	22:29	intron	-			D
TLR3	a,b,c	2.6E-10	rs3775291	4:186	missense	+	Caturegli et al. (2007)	Fine-map	D
TRMO	a,b,c	6.1E-06	rs8140060	9:97	intron	-			D

^a^FG, FinnGen.

^b^ AID, autoimmune disease.

^c^ Pub, Specific publication.

^d^ Risk, increasing (I) or decreasing (D) risk for hypothyroidism.

Table 2 also shows genes associated with “Hypothyroidism (congenital or acquired) (38.6 k cases, 263.7 k controls; 122 genes, phenotype a), “Hypothyroidism, strict autoimmune” (33.4 k cases, 227.4 k controls, 105 genes, phenotype b), and a more general term of “Disorders of the thyroid gland” (45.5 k cases, 263.7 k controls, 80 genes, phenotype c). Further validation is based on the independent cohort of 23&me (Eriksson et al., 2012). Several of the replicated genes were supported by fine-mapping (TLR3, Supplementary Material S3: Supplementary Figure S2).

We further extended the validation according to GWAS analysis from Taiwan, reporting on about 2,700 cases and ∼32,000 controls (Traglia et al., 2017). A collection of 824 variants (assigned to 66 coding genes) were associated with hypothyroidism. We identified 18 overlapping genes (23%) with PWAS gene list, and 26% with coding GWAS (Supplementary Material S2: Supplementary Table S8). We conclude that hypothyroidism in different genetic populations probably share similar genetic mechanisms for hypothyroidism.

### 3.7 Autoimmunity-associated genes are enriched in PWAS results for hypothyroidism

**FIGURE 6:**
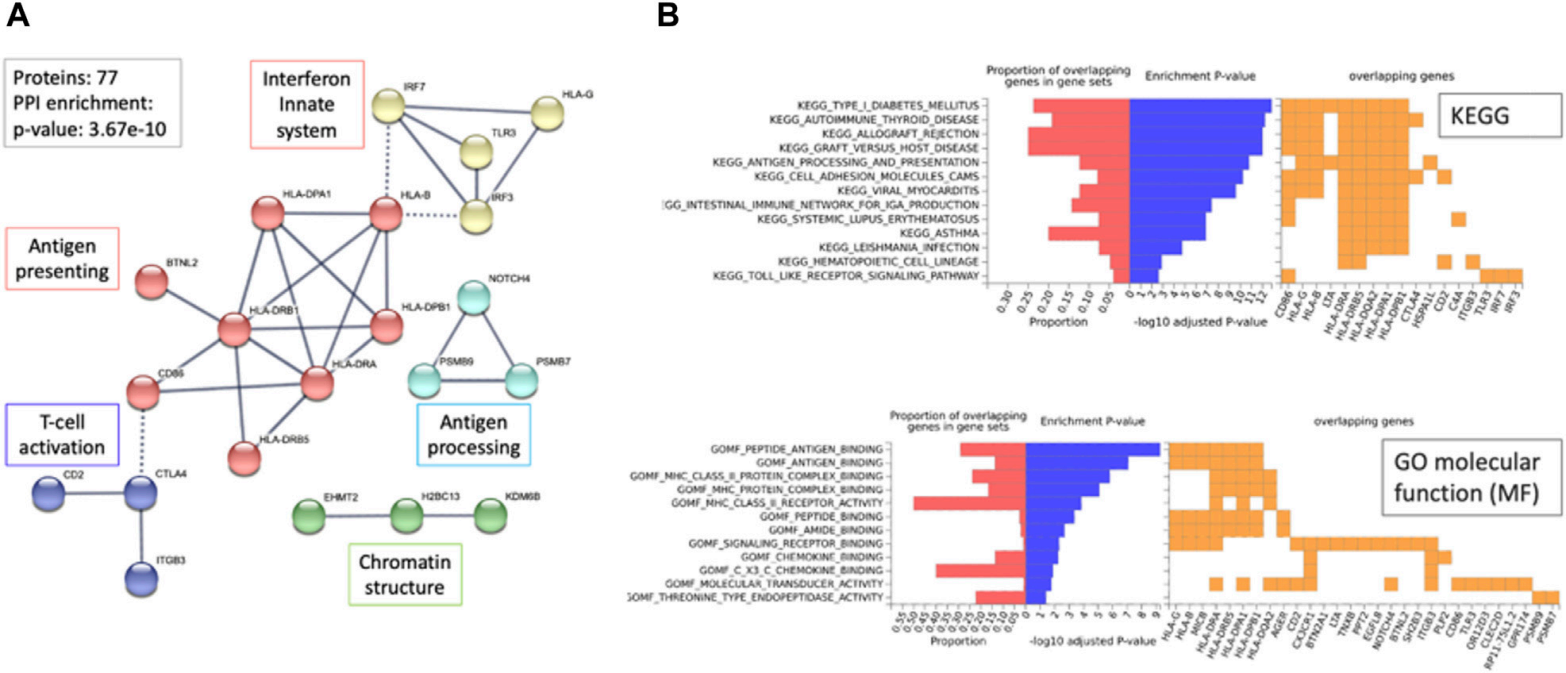
Network relationship and functional enrichment of PWAS results (77 genes). **(A)** The STRING network represents the genes connected at an interaction score >0.9. Dashed lines mark the connections between clusters. The unified function for each cluster is colored and annotated (e.g., antigen processing). **(B)** Enrichment analysis using the FUMA-GWAS Gene2Func protocol. In red, the fraction of genes in the gene set; blue, the adjusted *p*-value; orange, the overlapping genes for each term. The top 13 KEGG pathways and bottom, the GO_MF annotations. Note the enrichment of MHC genes (HLA-DPA1, HLA-DRB1, HLA-DRB5, HLA-B, HLA-DPA1, HLA-G) in KEGG and GO-MF analyses.

We asked whether the identified PWAS genes could highlight on the underlying mechanisms for hypothyroidism. To this end, we reconstructed a connectivity map among the 77 PWAS genes as represented by STRING (Szklarczyk et al., 2021) (Figure 6A).

The connectivity map is statistically significant and of high confidence (*p*-value 3.68e-10; with a PPI STRING score >0.9). The network (21 nodes) is mostly associated with cellular immunity, including antigen presentation, processing, and T-cell regulation. Moreover, 36 of 77 genes (47%) are located at the Chr6p22.1-p21.32 locus that specifies the MHC locus (hypergeometric distribution test, *p*-value 7.3e-57).

Relationships between MHC variants involved in autoimmunity determine diverse aspects of immunity, such as responses to infectious diseases and inflammation (Figure 6A). Extreme enrichment in coding genes identified within the MHC locus (Figure 6B, >60-fold higher than expected) strongly argues for the dominant genetic signal that combines hypothyroidism and autoimmune complex diseases. The enrichment observed in Gene ontology (GO) sheds light on the relevance of the genes involved in antigen recognition, chemokine binding, and multiple aspects of autoimmunity as revealed by the functional enrichment of KEGG pathways (Figure 6B).

### 3.8 Open Targets (OT) highlights the genetic basis of congenital hypothyroidism

**FIGURE 7:**
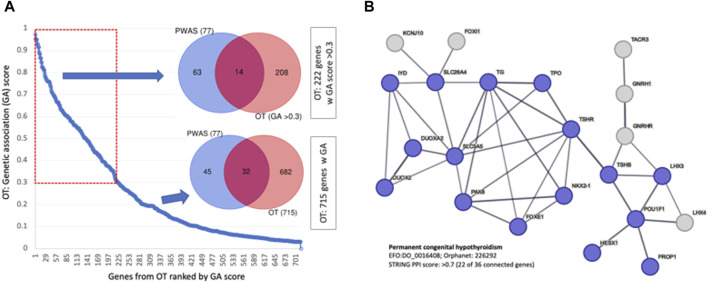
Genetic association with GWAS compiled by OT. **(A)** Ranked genes by their genetic association (by GA score, total of 715 genes). The overlap of 77 PWAS genes and 222 OT genes with GA scores >0.3 for all 715 genes. **(B)** A network relation of genes that are ranked by the OT global score >0.5 for the phenotype of permanent CH (total 36, 22 are connected, STRING PPI confidence score >0.7). The nodes are colored according to the match with the findings of CH causal genes from independent cohorts from India (Kollati et al., 2020) and China (Wang et al., 2020).

The OT platform provides a knowledge-based resource that converts the association of genes to diseases by including rich biological knowledge from multiple sources (e.g., literature, animal models, pathways, and drugs). Altogether, over 700 genes were scored by their genetic association (GA score ranges 0–1.0, see Methods; Supplementary Material S2: Supplementary Table S6). We estimated the significant of the overlap gene sets of the GA-list and PWAS. Using the cumulative distribution function (CDF) of the hypergeometric distribution showed that it is highly significant (*p*-value 6.38e-25; 10.5-fold enrichment). A stronger fold enrichment was observed for the subset of genes selected with higher GA score (total top 222 genes with score >0.3, *p*-value 5.28e-13, 14.7-fold enrichment; Figure 7A). Inspecting the functions of these 222 genes revealed many enzymes, membranous receptors, secreted proteins, and genes that act in the development, synthesis, and secretion of thyroid hormones. Surprisingly, 58% of the PWAS genes were not identified by the GWAS results reported by OT. Interestingly, none of the top-ranked genes for “hypothyroidism” according to the OT global score (25 genes; Supplementary Material S2: Supplementary Table S6) were identified as significant by PWAS.

We confirmed an extreme overlap with 19 of these top 25 genes as a CH-exclusive gene set, defined as “permanent congenital hypothyroidism” (Orphanet: 442; EFO: 0,016,408). Figure 7B shows that the genes associated with permanent CH are functionally linked (STRING enrichment, *p*-value <1e-16). Validation of the CH causal genes was confirmed by independent studies analyzing patients from Korea (Jung et al., 2020) and China (Wang et al., 2020) (colored blue, Figure 7B).

### 3.9 Gene-based association studies by sex

**FIGURE 8:**
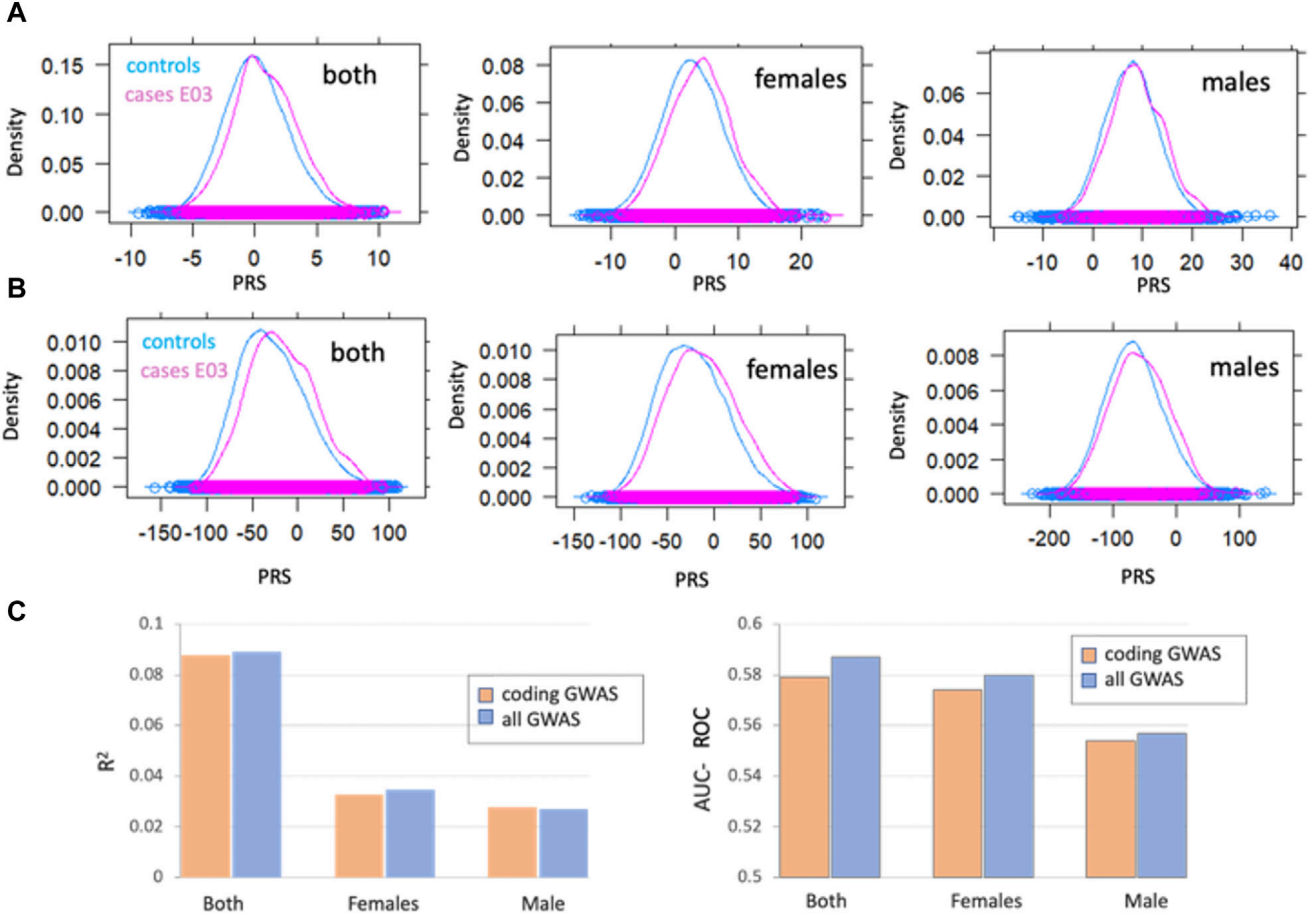
Gene-based association analysis by sex **(A)** Distribution of a polygenic risk score (PRS) among individuals with and without E03 diagnosis, marked as cases (pink) and controls (blue). PRS scores were calculated for all GWAS **(A)** and coding-GWAS **(B)** for the entire cohorts (both), females and males. **(C)** PRS prediction by the coefficient of determination (*R* ^2^, left) AUC-ROC (right) for coding GWAS (orange) and all GWAS (blue) for the entire cohort (both) and by sex. Coding GWAS variants partitioned by sex are listed in Supplementary Material S2: Supplementary Table S9.

Following filtration of the UKB population (see Methods), there were 2,557 males (19%) and 11,094 females (81%) with high quality genotyping data and E03 diagnosis. The strong sex imbalance of ICD-10 E03 raised the question of whether hypothyroidism is signified by sex-dependent genetics. To this end, we applied the PWAS gene-aggregative approach separately for males and females (Figure 8).

The polygenic risk score (PRS) was calculated as a weighted sum of allele dosages multiplied by their corresponding effect sizes for females, males and both groups. PRS reflects the cumulative effect of the genetic variants, thus allows for predicting individual predisposition for hypothyroidism. Figure 8A (all GWAS) and Figure 8B (coding GWAS) show the distribution for the entire population and, according to the sex partition. Figure 8C shows the prediction power of the PRS as calculated by the *R* ^2^ and AUC-ROC for the test set (set aside 2,492 cases and 51,630 controls). We conclude that the majority of the PRS predictive power is captured by variants within the coding regions. Moreover, the separation of the population by sex validated that most genetic signals for the calculated AUC-ROC were captured within the female group.

## 4 Discussion

In this study, we sought to identify the genetic basis of hypothyroidism (ICD-10, E03) in the adult population of the UKB. The complex origin of primary hypothyroidism is associated with the differentiation between congenital and acquired conditions. Moreover, diseases such as Hashimoto’s thyroiditis and Graves’ disease (GD) are linked to hypothyroidism through the immune system. Other forms of hypothyroidism may be associated with organ resistance to thyroid hormone (Persani et al., 2010). Although the elevated prevalence of the condition in older females is well-established (Dunn and Turner, 2016), the underlying genetics is only partially resolved.

In this study, we exhaustively compared different association study methods and protocols. In a routine GWAS, variants are statistically tested within case‒control setting under and the additive model (Tam et al., 2019). However, PWAS also detects non-additive effects and allows the aggregated effect of variants that may occur at different locations within the same gene (i.e., compound heterozygosity). Although only a small fraction of the PWAS identified genes have been identified with a clear recessive signal (Supplementary Material 2: Supplementary Table S5, Supplementary Material S3; Supplementary Figure S1), such inheritance modes have been mostly overlooked by routine GWAS approaches (Edwards et al., 2013; Tam et al., 2019). The vast majority of the associated variants in GWAS occur in the noncoding regions of the genome, and thus, the relevance of a SNP function to the studied phenotype is mostly lacking [discussed in (Edwards et al., 2013)]. Along with the effort to capture gene causality due to regulation, TWAS was developed to identify variants that affect gene expression level (e.g., eQTL) using tissue-based data and Bayesian considerations. Significant variants from TWAS are located in regions that may alter the expression levels of transcripts, with cis or trans regulation modes. As expected, TWAS exposed quite different results relative to PWAS or OT compilation of GWAS findings (Figure 5B). Much of the functions of genes identified by TWAS and or by the OT are linked to thyroid development and hormone secretion (e.g., PAX8, FOXE1, STAT4, TG). Interestingly, TWAS and OT display a minimal signal of immunity (Khan et al., 2021).

The genetic basis for congenital hypothyroidism (CH) was exposed from population studies, where dysregulation of transcription factors (TFs) characterizes individuals with CH (Grasberger and Refetoff, 2011). A comprehensive screening of CH in family pedigrees from China identified DUOX2 as the most frequently mutated gene (Sun et al., 2018). CH, combined with neonatal diabetes mellitus, is caused by mutations in the TF GLIS3 gene (Fu et al., 2018). Other TFs, such as NR1D1 and PAX8, which are exclusively expressed in thyroid cell types, were identified by GWAS and TWAS. TFs such as FOXE1 and STAT4 were consistently identified by all large-scale GWAS. These genes act during embryogenesis to establish the pituitary, hypothalamus, and thyroid axes (Table 1). Thyroid signaling were highly represented by TWAS. For example, PDE8B is expressed primarily in the thyroid to execute the TSH effects.

Combining results from multiple classical GWAS identified TFs that have strong links to thyroid function (e.g., NKX2-1; NK2 homeobox 1). It is likely that the strong effect size of rare variants dominated their discovery (see OMIM lists of 34 genes for CH (Amberger et al., 2019), Supplementary Material S2: Supplementary Table S10). None of the top-scoring OT genes were identified by PWAS. We attribute this discrepancy in gene findings to the relatively small effect sizes of the most common variants in coding genes. Recall that GWAS identifies strong functional elements that are ignored by PWAS. For example, a cluster of variants on chromosome 9, including rs10759927, rs7850258 and rs7030280, was significantly identified by classical GWAS for hypothyroidism (with a *p*-value ranging between 1e-82 and 2e-100). These variants occur within the introns of PTCSC2, a noncoding RNA (ncRNA) that is expressed exclusively in the thyroid. Furthermore, accumulated data propose that CH is not restricted to monogenic dominant mutations. Biallelic effects were suggested based on the sporadic occurrences of CH within families (Fu et al., 2018). Additionally, evidence of parents with clinical manifestations of thyroid (functional or morphological) supports the notion of predisposition with recessive inheritance (Leger et al., 2002). In this study, we have not explicitly studied rare variants from whole genome or whole exomes (Backman et al., 2021). We argue that applying a recessive model in PWAS and including a comprehensive analysis of rare variants can expose overlooked genetics with pathological variants that display relatively strong effect size.

The strongest signature of explainable hyperthyroidism is caused by dysregulation of the immune system. This signature was revealed by applying PWAS and, to a lesser extent, coding GWAS. Testing the genetic variations associated with thyroid autoimmunity identified a strong interaction with pathways driving the immune response (Khan et al., 2021; Luo et al., 2021). The findings agree with a clinical investigation that explains the mechanisms underlying thyroid autoimmunity. Hashimoto’s thyroiditis is the most common form of hypothyroidism. The autoimmune facet of hypothyroidism is characterized by the infiltration of T lymphocytes into the thyroid gland and autoantibodies against thyroid-specific genes (e.g., thyroid peroxidase, thyroglobulin, and TSH receptor). AIT-associated genes were also identified in thyroid autoimmune Graves’ disease (GD). Among these shared genes are HLA class II (HLA-DR), protein tyrosine phosphatase 22 (PTPN22), and cytotoxic T lymphocyte antigen 4 (CTLA4) (Jacobson and Tomer, 2007). The genetic signals of autoimmune thyroiditis are shared with other immune-mediated diseases, such as T1D, celiac disease, rheumatoid arthritis (RA), systemic lupus erythematosus (SLE), and psoriasis. PWAS exposes many immune-related genes that carry coding variants (e.g., PTPN22, SH2B3, and genes in the class 1 MHC region). Thus, it can help to assess the risk prediction for autoimmunity that overlaps with hypothyroidism (Eriksson et al., 2012). Importantly, for a large collection of common diseases (Brandes et al., 2020; Brandes et al., 2021; Zucker et al., 2023), we found no evidence for associations within the Chr 6 MHC locus by PWAS or coding GWAS. Therefore, we argue that an immune-related signature is a genuine contributing signal for hypothyroidism with gene-specific interpretability.

The gene-based analysis performed in this study raised the question of whether genetic effects are distinguished by sex. For hypothyroidism, a ratio of >3.6:1 for females and males was reported in the UKB. This bias in prevalence was also validated in the Finnish population, which reported 37,942 affected females and 9,616 males. Clinical observations indicate the relevance of sex-dependent risk assessment for E03. The average age of diagnosis for females and males was different in UKB (50 and 58 years for females and males, respectively). Sex also was shown to vary the calculated risk. For example, the occurrence of multiple variants for CTLA4 gene in the Taiwan cohort increased the risk for hypothyroidism (odds ratio (OR) 1.4–1.9). But, the risk was much higher for overweigh males (OR 4.4–5.3) with such CTLA4 variants (Liu et al., 2022).

In this study, we showed that the sex stratification of hypothyroidism provided no support for genuine genetic differences between the sexes, despite the large gap in prevalence. This agrees with most human traits and diseases that do not support a mechanistic difference between the sexes (Traglia et al., 2017). We recently showed that for primary hypertension (ICD-10 I10) and phenotypes of blood pressure most of the genetic signal was attributed to females, despite a higher prevalent in males (Zucker et al., 2023). With the continuous increase in statistical power due to cohort sizes, more cases of sex-dependent genetics have been revealed (Pirastu et al., 2021).

Notably, the presented pipeline is applicable to other complex diseases with unresolved etiology. Nevertheless, the association methods used in this study are sensitive to cohort size. Therefore, the methods are mostly suitable for diseases that are signified by relatively high heritability and sufficient cohort size. Another limitation concerns the fact that while most variants occur outside of the coding region in regulatory regions, PWAS, and coding GWAS are restricted solely to coding regions and primarily to common variants (Brandes et al., 2022). Furthermore, this study focuses on Caucasian-white ethnicity. It would be of great importance to apply the pipeline on populations of other origins.

We conclude that comparison results that rely on capturing different aspects of the genetic signal allowed us to reveal the complex etiology of hypothyroidism, covering recessive signals, CH, and acquired chronic damage to thyroid functionality. The discovery benefited from the use of complementary association studies and utilizing the same set of variants for comparison. We suggest that the framework is applicable and can be generalized to other polygenic diseases with unknown etiologies. It is likely that complex diseases for which the age of diagnosis, sex prevalence, and diseases that are subjected to (wrong) alternative diagnoses could benefit from integrating genetic association schemes. Moreover, using coding GWAS and PWAS, we illustrate the clinical benefits of gene-based genetics by improving interpretation, which can benefit unexplored therapeutic targets.

## Data Availability

The data analyzed in this study was obtained from the UK Biobank, the following licenses/restrictions apply: individual application required. Requests to access these datasets should be directed to the UK Biobank: access@ukbiobank.ac.uk.

## Abbreviations

AIT, autoimmune thyroiditis; AUC-ROC, Area under the receiver operating characteristic curve; CH, Congenital hypothyroidism; GA, Genetic association; GD, Graves’ disease; GWAS, Genome wide association study; HPT, hypothalamic-pituitary-thyroid; HWE, Hardy-Weinberg equilibrium; LD, linkage disequilibrium; MAF, Minor allele frequencies; MHC, Major histocompatibility complex; ncRNA, non-coding RNA; OMIM, Online Mendelian Inheritance in Man; OT, Open Targets; OTG, Open Targets Genetics; PPI, Protein-protein interaction; PRS, polygenic risk score; PWAS, Proteome wide association study; SNP, Single nucleotide polymorphism; TWAS, Transcriptome wide association study; UKB, UK Biobank.
