# Supplementary material for: Revealing the genetic complexity of hypothyroidism: integrating complementary association methods
Source: Front Genet. 2024 Jun 11;15:1409226. doi: 10.3389/fgene.2024.1409226 (PMC11196612; doi:10.3389/fgene.2024.1409226)
Supplement: Supplementary file 2 [file Table1.DOCX]

**Functional Insights into Hypothyroidism Etiology through Complementary Genetic Association Methods**

**Roei Zucker, Michael Kovalerchik, Amos Stern, Hadasa Kaufman, Michal Linial**

**Supplementary data**:

**Additional file 1: Supplementary Text S1.**

**Additional file 2: Supplementary Tables S1-S9.**

**Additional file 3: Supplementary Tables S1-S9.**

**Additional file 1: Supplementary Text S1.**

**Mapping UniProt ID to RefSeq**

The coding GWAS analysis includes the human proteome according to UniProt-SwissProt (labeled “reviewed”). Due to the unambiguous mapping of RefSeq gene names, we cover 18,053 protein-coding genes of the ~20k proteins that are listed for the human proteome. The missing genes are instances where the mapping of genes to proteins is ambiguous (e.g., genes with multiple protein versions). In addition, no mapping is available to nongenuine RefSeq genes such as endogenous retroviruses (e.g., ERVK) or the constant and variable regions of immunoglobulins (e.g., IGLV and IGKV). The 2,119 UniProtKB-reviewed gene names that are not included in the PWAS analysis are listed in Supplementary file 1: **Table S1**. Due to the relative scarcity of variants in the coding GWAS (c-GWAS) analysis, we excluded any MAF filtration, and applied HWE threshold of 1e-10 and Geno 0.1. Altogether, out of the overall ~97 M variants, we tested all 639,323 coding and splicing variants located within 18,053 protein-coding genes.

**GWAS summery statistic**

We extracted the list of associated variants from OpenTargets genetics (OTG) for comparative analysis from six large-scale GWAS for associated phenotypes. The cohorts from these GWAS ranged from 250k to 580k controls and 15k to 33k cases. The number of independent loci in each GWAS is 115 (Neale v2, 2018), 45 (UKB Saige 2018), 65 [1], 160 [2], 133 [3] and 67 (FinnGen, Freeze 6, 2022). Note that the study of Neale’s lab (2018, V2) focused on European ancestry with 361,141 participants, among whom 17,574 were labeled with "hypothyroidism/myxedema (self-reported, noncancer)", while other GWAS from UKB were performed across multiple ethnic groups [2].

**Validation**

For validation, we used the 146 independent loci reported in Freeze 7.0 (Fz7; spring 2021). Fr7 lists of credible genes but includes loci that failed to identify creditable genes (33 loci). Note that for other loci, several genes have been reported. Altogether, there were 102 genes for E4_HYTHYNAS (hypothyroidism, other/unspecified) from 38,554 cases and 263,704 controls. Additional validation was performed for FinnGen Freeze 8.0 (Fz8, spring 2002). We tested the related phenotypes of E4_HYTHY_AI_STRICT (Fz8: 45,320 cases) and E4_HYTHYNAS (Fz8: 52,828 cases). We compared the results for PheWAS Section 4 (i.e., endocrine, nutritional and metabolic diseases, E4) analyzed by the Ristey R9 querying system [4].

**References**

1. Sakaue S, Kanai M, Tanigawa Y, Karjalainen J, Kurki M, Koshiba S, Narita A, Konuma T, Yamamoto K, Akiyama M *et al*: **A cross-population atlas of genetic associations for 220 human phenotypes**. *Nat Genet* 2021, **53**(10):1415-1424.

2. Donertas HM, Fabian DK, Valenzuela MF, Partridge L, Thornton JM: **Common genetic associations between age-related diseases**. *Nat Aging* 2021, **1**(4):400-412.

3. Kichaev G, Bhatia G, Loh PR, Gazal S, Burch K, Freund MK, Schoech A, Pasaniuc B, Price AL: **Leveraging Polygenic Functional Enrichment to Improve GWAS Power**. *Am J Hum Genet* 2019, **104**(1):65-75.

4. Kurki MI, Karjalainen J, Palta P, Sipilä TP, Kristiansson K, Donner KM, Reeve MP, Laivuori H, Aavikko M, Kaunisto MA: **FinnGen provides genetic insights from a well-phenotyped isolated population**. *Nature* 2023, **613**(7944):508-518.

**Additional file 2: Supplementary Tables S1-S10.**

**Table** **S1.** A list of protein coding genes that are not included in PWAS analysis (total 2119).

**Table S2.** Summary statistics for hypothyroidism comparative study by GWAS from OT.

**Table S3.** GWAS for gene length (g-GWAS) for hypothyroidism with gene annotation.

**Table S4.** Results of coding GWAS (c-GWAS) with their associated significant genes.

**Table S5.** Results of PWAS with significant genes by their effect size and heritability modes.

**Table S6.** A sorted list of >700 genes identified by genetic associations from OT.

**Table S7.** Results from TWAS (UTMOST) protocol from webTWAS.

**Table S8.** Results from Venn diagrams for the different association studies.

**Table S9.** Table of significant sex-dependent coding-GWAS with gene names.

**Table S10.** A list of genetic related genes for CH according to OMIM.

**Additional file 3: Supplementary Figures S1-S3.**


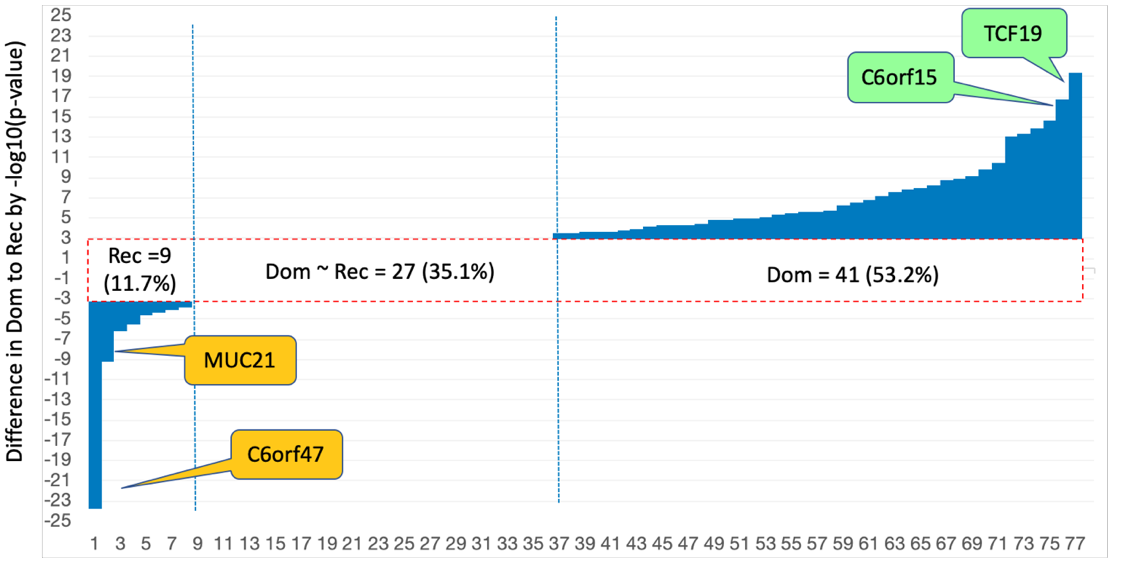


***Figure S1.*** *PWAS genes. Difference in the statistical significance measured with a recessive and dominant inheritance model for each of the 77 identified genes (PWAS FDR <0.05; Rec and Dom, respectively). The dominant inheritance model only requires a single allele to be affected, while for the recessive inheritance model, both alleles should be affected so that gene functionality will be affected. We set the difference in -log10(p-value) to be at least 3 to depict a heritability preference. The symbol of two most significant genes for the recessive (yellow) and dominant (green) models are indicated.*


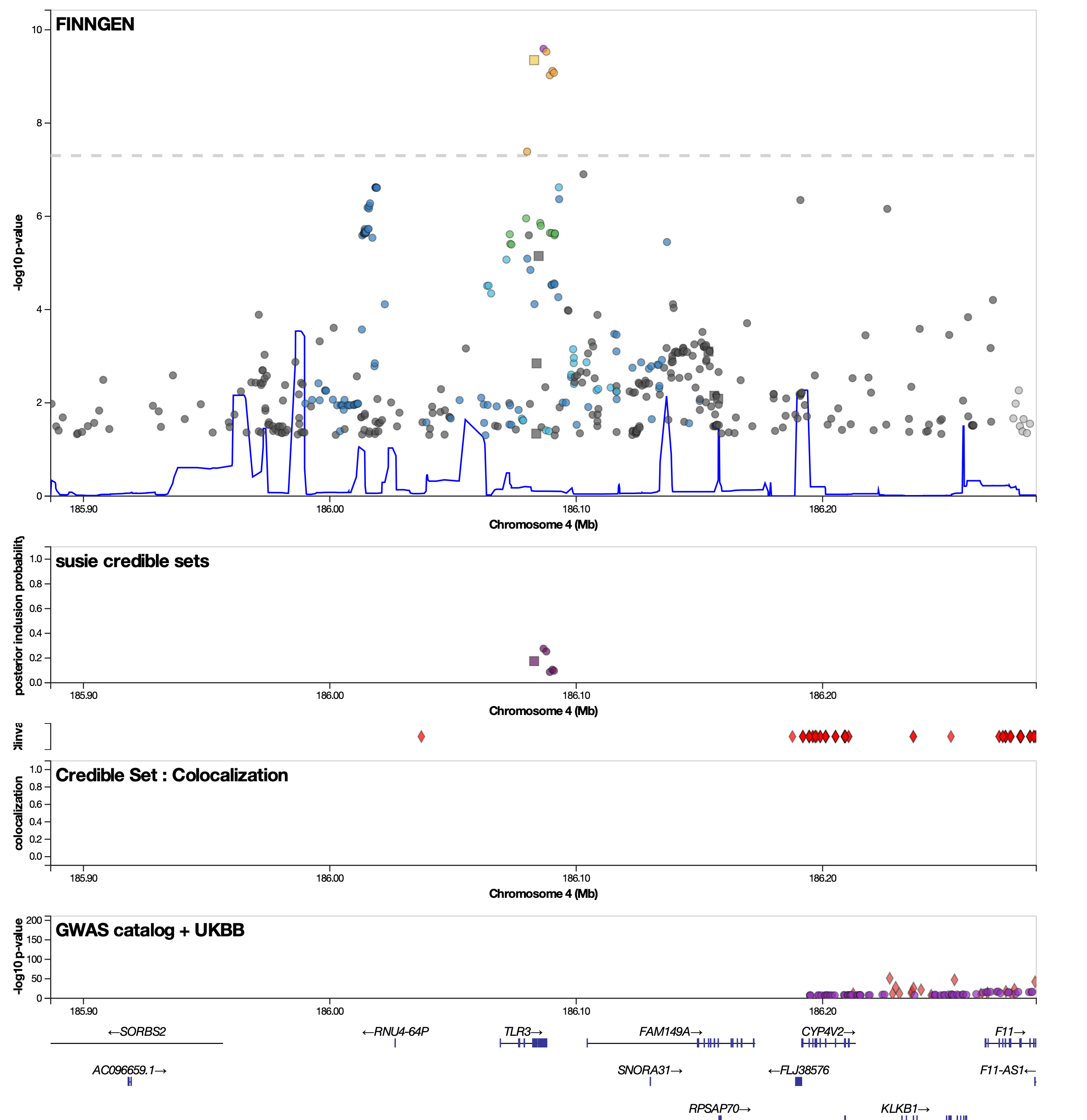

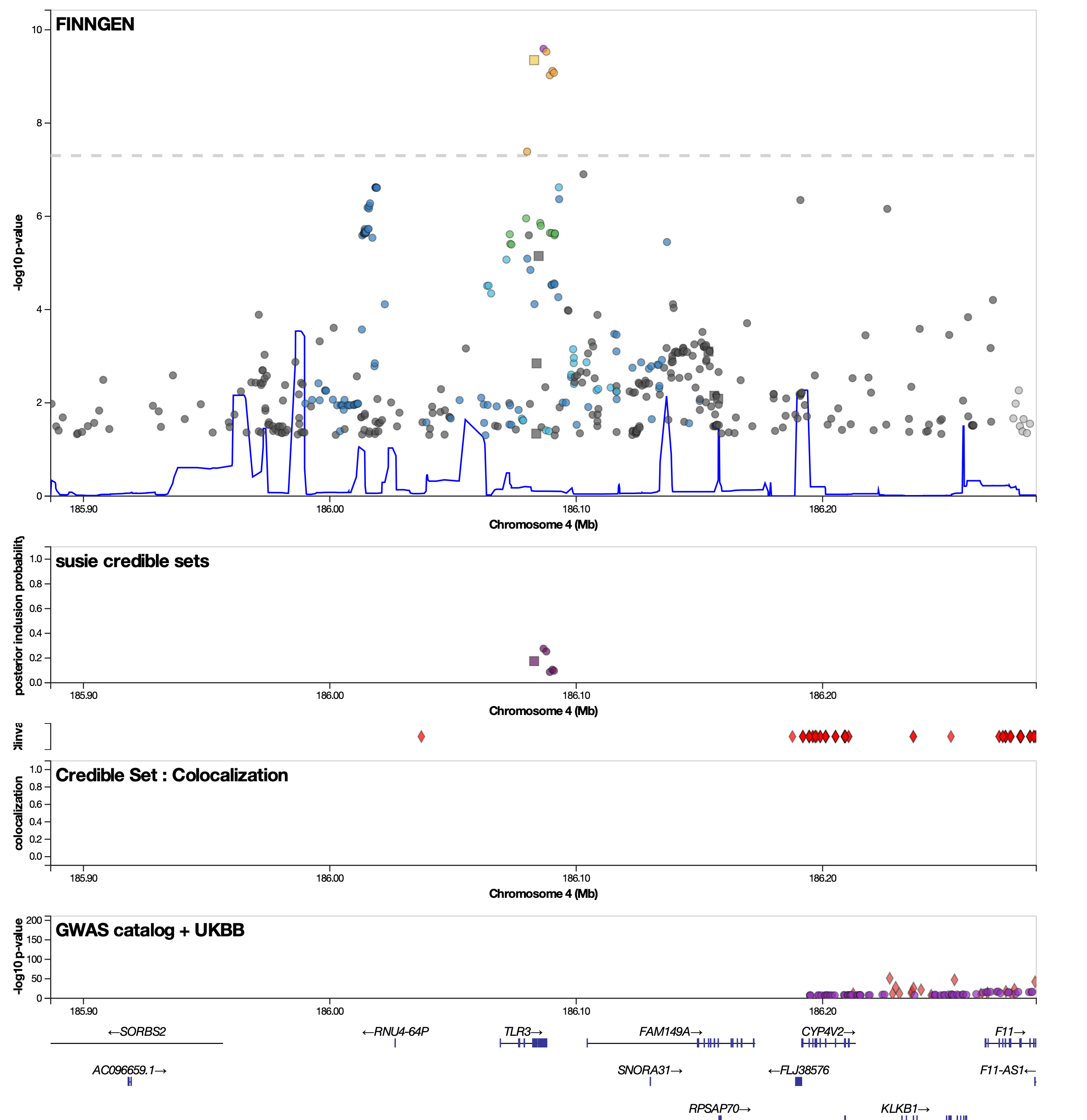


***Figure S2.*** *Fine mapping of loci from Chromosome 4 from 185.9 to 186.3 Mbp. TLR3 is the candidate causal gene as indicated by the red frame. Data is extracted from FinnGen Fz7.*

**
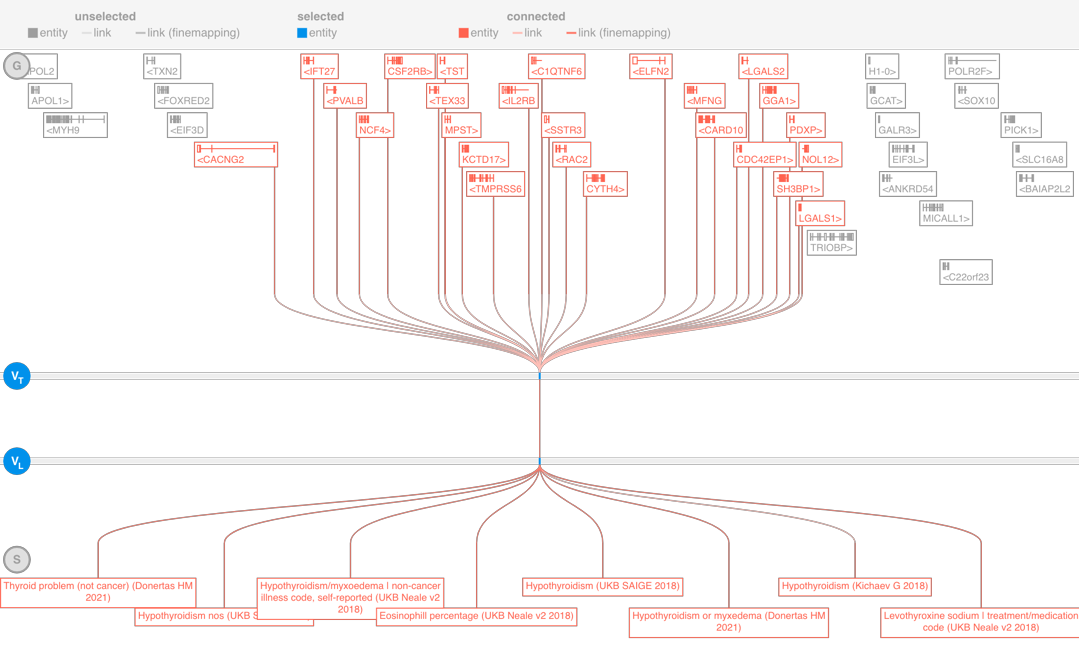
**

***Figure S3.*** *Loci around the gene C1QTNF6 (C1q and TNF related 6) that is located in the vicinity of >20 genes that could not be determined explicitly as causal. Note that genes within the loci are associated*

*with hypothyroidism and several thyroid-specific phenotypes.*
